# Dynamical landscapes of cell fate decisions

**DOI:** 10.1098/rsfs.2022.0002

**Published:** 2022-06-10

**Authors:** M. Sáez, J. Briscoe, D. A. Rand

**Affiliations:** ^1^ The Francis Crick Institute, 1 Midland Road, London NW1 1AT, UK; ^2^ IQS, Universitat Ramon Llull, Via Augusta 390, Barcelona 08017, Spain; ^3^ Mathematics Institute, University of Warwick, Coventry CV4 7AL, UK; ^4^ Zeeman Institute for Systems Biology and Infectious Epidemiology Research, University of Warwick, Coventry CV4 7AL, UK

**Keywords:** cellular decision-making, Waddington landscape, development, dynamical systems, bifurcations

## Abstract

The generation of cellular diversity during development involves differentiating cells transitioning between discrete cell states. In the 1940s, the developmental biologist Conrad Waddington introduced a landscape metaphor to describe this process. The developmental path of a cell was pictured as a ball rolling through a terrain of branching valleys with cell fate decisions represented by the branch points at which the ball decides between one of two available valleys. Here we discuss progress in constructing quantitative dynamical models inspired by this view of cellular differentiation. We describe a framework based on catastrophe theory and dynamical systems methods that provides the foundations for quantitative geometric models of cellular differentiation. These models can be fit to experimental data and used to make quantitative predictions about cellular differentiation. The theory indicates that cell fate decisions can be described by a small number of decision structures, such that there are only two distinct ways in which cells make a binary choice between one of two fates. We discuss the biological relevance of these mechanisms and suggest the approach is broadly applicable for the quantitative analysis of differentiation dynamics and for determining principles of developmental decisions.

## 1. Introduction

How the diversity of molecularly and functionally distinct cell types that comprise a living organism arise during embryogenesis is a central concern of developmental biology. The process is progressive. As cells proliferate and assemble into tissues, their molecular identity changes in discrete step-like transitions to produce diverging sequences of distinct cell states that culminate in the differentiation of specific functional cell types. Hence, cellular development can be viewed as sets of branching cell lineages generating increasing diversity and comprising increasingly specialized cell types. This is directed by intercellular signalling between differentiating cells making the process non-autonomous and self-organizing. Each branch-point in a cell lineage represents a choice between alternative distinct cell types. The choice a cell makes at each transition is referred to as a *cell fate decision*.

This process was pictured by Conrad Waddington in the 1940s and led to his famous 1957 drawing of the epigenetic landscape [1]. In his iconic image, the differentiation trajectory of a cell is conceived as a ball rolling down a landscape of branching valleys. At watersheds, the valleys branch and the ball must decide between one of two available paths, representing alternative cell fates. This view has had an enormous influence on the field, but efforts to convert the metaphor to models that make quantitative, experimentally testable predictions have proved challenging. It is now clear that extrinsic signals organize cell fate decisions in developing tissues by regulating the gene expression programme and therefore the functional properties of cells.

The signals and the downstream transcriptional responses form dynamic and complex circuits, termed gene regulatory networks (GRNs). In this way the activity of the GRN controls the location and timing of cell fate allocation in a tissue. Reconciling this view of development with that of Waddington has led to the idea that GRNs and their controlling signals create the landscape and the route a cell takes through it.

Mathematical models have been used extensively to investigate how the dynamics of GRNs regulate cell fate decisions. These have offered insight into how features such as switch-like responses and feedback control regulate cell fate decisions, and support the idea that cell fates can be represented as the steady states of a multistable GRN. However, these models require bespoke case-by-case construction and expand rapidly in size and complexity as more genes and interactions are included. Moreover, information about their structure and parameters is often difficult to obtain and the resulting complexity can obscure underlying mechanisms. Nevertheless, the fundamental framework of cellular decisions described by a GRN model remains relatively simple: cells transition between a limited set of discrete cell fates. Hence, methods to represent cell dynamics and account for transitions between states in a way that classifies cell decision behaviours would complement existing approaches and provide insight into the underlying principles. Here we discuss recent ideas that recast Waddington’s landscape metaphor in the context of a dynamical theory of cellular decision-making [2]. The aim is not just to describe the theory but to use this to provide a practicable approach to the analysis of gene expression data in differentiating cells that will allow the elucidation of decision-making structures.

Tools to construct models that describe dynamics quantitatively as well as qualitatively depend on mathematical developments initiated by Smale [3] in the 1960s, together with the insights arising from Thom’s catastrophe theory [4–7]. This has two important consequences for comparisons with Waddington’s landscape metaphor. First, the intuition that cell states flow downhill following a trajectory defined solely by the landscape’s topography given by a height function, is an oversimplification. As we explain below, such a topography does not uniquely define the dynamics. For decision-making, it is necessary to understand the topology of the connections between cell states determined by certain invariant manifolds. The height function that describes the landscape does not determine these without extra information (see box 2).

**Figure 1.**
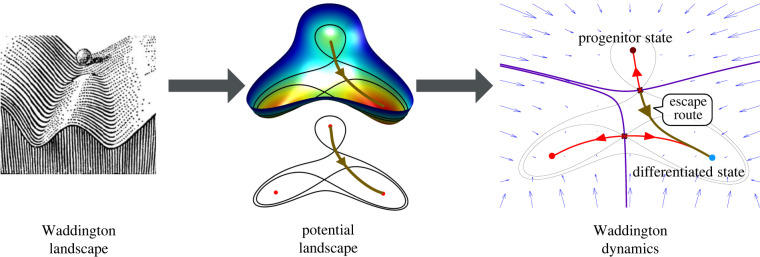
From Waddington’s landscapes to Waddington dynamics. The classic picture of a Waddington landscape (left) can be formalized using a potential function (middle). Nevertheless, to capture the full behaviour of the system complete Waddington dynamics are needed (right). The dynamics describe the trajectories cells take between different states.

We refer to this approach, which includes the connection topology, as *Waddington dynamics* (figure 1). An important aspect of the approach we describe is the idea that the connections between cell states along which transitions take place correspond to the unstable manifolds of certain saddle points sitting between the system’s attractors. Thus, our discussion includes a precise idea of a *transition state* (see §5.1).

Second, the focus on Waddington dynamics points to another important issue. Until recently, discussions about decision-making in Waddington landscapes emphasized the so-called *local bifurcations* in which a decision results from a change confined to a small region of the landscape (disappearance or appearance of a state). This neglected *global bifurcations* that alter the dynamics in ways that are not constrained to a small region (by changing the routes that cells take). Recent work has indicated that global bifurcations can play an important role in cell fate decisions [8–11].

Despite these complications, adopting this dynamical systems perspective has several advantages. The models that represent cellular decisions are highly constrained. This provides a classification scheme for cell fate decisions that is independent of the molecular details of the underlying GRNs. This reveals a small number of decision archetypes and suggests underlying design principles. In turn, this leads to a practicable approach for using experimental data to construct quantitative models that make testable predictions [8–11].

In the following sections, we introduce the mathematical concepts necessary to construct and interpret these types of geometric models. We argue that these can be used to develop new tools to interpret the type of single-cell expression data that is becoming available. Moreover, these tools provide insight into the underlying principles and point the way to discovering mechanism. An outline of an experimental and analytical strategy for applying this approach is provided (box 2). We address two obvious problems about GRN-centred approaches derived from the genome-wide nature of data produced by technologies such as scRNAseq. The first is the much discussed curse of dimensionality. The second is the complexity of the interactions between the different molecular components that makes both textual and mathematical description difficult. Our approach tackles these problems as the structure of the landscape is described independently of the precise molecular details and is agnostic to dimension.

## 2. Motivating a dynamical systems approach: modelling and experiments

To illustrate relevant dynamical systems concepts, we start by discussing two decision-making scenarios where cells in a precursor state *P* choose to differentiate to one of two downstream (more differentiated) states *A* or *B*. This type of decision is common in developmental systems. We present in this section the rationale we used in [11] to build a mathematical model for such a decision-making system which allowed the informed design of experiments.

**Figure 2.**
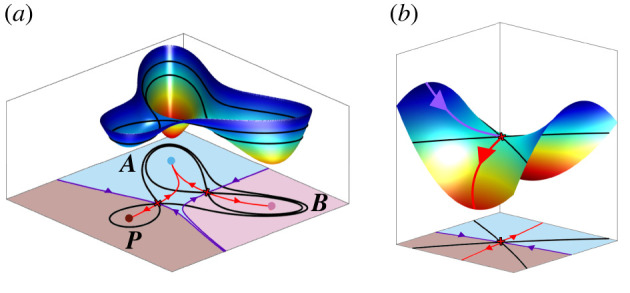
Two-dimensional landscapes. (*a*) System with three attractors and two saddles. The purple curves are the separatrices or stable manifolds of the saddles, the red curves are the unstable manifolds (if time is run backwards points on these converge to the saddle). Points on each unstable manifold converge to two attractors and the stable manifolds divide the phase space into three basins, one for each attractor. Level curves for the saddles are indicated in black. (*b*) Saddle point. The dynamics near a saddle point. The stable (purple) and unstable (red) manifolds are shown.

In these examples (and more generally), the dynamical flow can be pictured on a two-dimensional (2D) surface where every point has a specific height, given as a function of its position by a so-called *landscape potential function* (figure 2). The landscape contains well-like depressions which we call *basins* by analogy with the basin of a river. These basins are separated by intervening ridges (figure 2*a*). We can think of the dynamics of a differentiating cell as being described by a ball rolling downhill. If it starts in a basin, it will roll to the bottom of the basin, where the potential function is minimal. In the language of dynamical systems, this point is an *attractor* and in the developmental context it corresponds to a cell state. As in real landscapes, to move from one basin to another the intervening ridge has to be crossed. The point on the ridge that requires the minimal amount of climbing to traverse is called a *saddle* because of its shape (figure 2*b*). In a 2D landscape there may also be peaks. Trajectories flow away from these, so they are called *repellors*.

**Figure 3.**
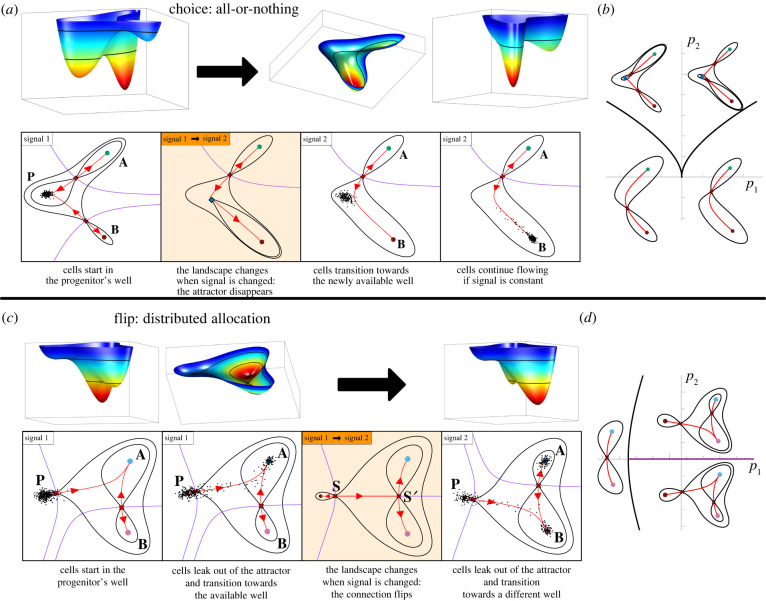
Binary decisions. (*a*) Binary choice landscape. In the starting condition, Signal 1, cells are in a progenitor state corresponding to the middle basin of the landscape. Exposure to Signal 2 results in the disappearance of the central basin and the bottom ridge and cells transition into State *B* following the slopes of the new landscape. (*b*) Two parameters *p*_1_ and *p*_2_ govern the landscape in (*a*). Bifurcations occur when these parameters are on the black curves in *p*_1_, *p*_2_-space. Crossing one curve results in cells transitioning to *A*; crossing the other, cells transition to *B*. (*c*) Binary flip landscape. Cells start in the progenitor state *P* which corresponds to a shallow basin. Noise is sufficient for cells to transition into the more committed states and they follow the escape route (red) towards state *A* when exposed to Signal 1. In response to Signal 2 the escape route flips towards *B* and cells transition towards *B*. (*d*) The landscape in C is also governed by two parameters. The progenitor attractor is destroyed when the parameters are on the black curve and the flip occurs when the parameters are on the purple curve.

In our examples, cells start in a precursor state, attractor *P*, and they decide between one of two successor fates *A* and *B*. Theoretical considerations indicate that there are essentially two ways for the decision to proceed: either an all-or-nothing case where all cells starting in the *P* attractor adopt the same fate, or a distributed allocation in which *P* cells adopt fates *A* and *B* with varying proportions (figure 3).

In the first case (figure 3*a*), three attractors are linearly arranged such that the precursor state attractor *P* sits between the two more committed states *A* and *B*. Connecting the three basins are two saddles that act as passes in the landscape. A cell fate decision corresponds to the disappearance of the *P* attractor in response to an inductive signal (shaded panel in figure 3*a*). This occurs when the inductive signal modifies the landscape so that the *P* basin gradually shrinks until the *P* attractor collides with one of the saddles (local bifurcation). This forces progenitor cells in *P* to flow down to one of the committed states, *A* or *B*. The connections between attractors (red curves in figure 3) remain unchanged. Which attractor is chosen is determined by which of the two saddles collided with *P*. This results in all cells exiting the *P* attractor adopting the same fate. Consequently, we call this the *binary choice* landscape. Figure 3*b* shows a schematic of this family of landscapes.

The second case has a different arrangement of basins and differs from the first in that it contains a global bifurcation (change in connections). As before there are two saddles in the landscape, but in this case, one saddle connects the two differentiated states *A* and *B* and, for almost all parameters, the other saddle connects the progenitor state *P* to either *A* or *B* (figure 3*c* red curves). As with the binary choice, cell transitions are a consequence of the destabilization of the *P* attractor. However, there is only one pass with which to collide and when the collision happens there is a well-defined *escape route* (connection between attractors) which determines the chosen differentiated state *A* or *B*.

A change in signals can alter this choice by causing the escape route to flip so that instead of leading to *A* it leads to *B*. This happens when the parameters cross the ‘flip line’ (dashed purple line figure 3*d*) and results in a change in the decision made by cells leaving *P* as a consequence of a change in the connections. On the flip line the trajectory leaving the upper saddle goes directly to the lower saddle (shaded panel in figure 3*c*).

When the escape route is close to the flip line, some noise in the system can result in wayward cells adopting the alternative committed fate. The closer the system is to flipping from one attractor to the other then the more equal will be the allocation of cells between the *A* and *B* states. Thus varying the relevant signals regulates the proportions of *P* cells adopting *A* or *B* identities. For this reason, we call this landscape structure the *binary flip landscape* (figure 3*d*).

These two landscapes (figure 3) highlight key features of the dynamics associated with decision-making. The basins and the saddles that connect them play a crucial role in geometric models. We will describe how these behave and point out that their structure is highly constrained. This in turn leads to a classification scheme of underlying landscape structures that form archetypal decision mechanisms. We will highlight the landscapes that we think are most likely to occur. The above two examples are the simplest of these. We suggest that these will be the most common wherever a progenitor state differentiates into two more-committed states.

We proposed that the binary choice landscape explains how mouse embryonic stem cells (ESCs) that have differentiated to an epiblast-like state decide whether to adopt either neural or caudal epiblast identity in response to WNT and FGF signalling [11]. A decision structure similar to the binary choice landscape has also been proposed to explain the differentiation of mouse blastocyst inner cell mass cells to either epiblast or primitive endoderm [12,13]. By contrast, we proposed that the flip landscape is responsible for the fate decision involved in allocated cells to either spinal cord or mesoderm identity during formation of vertebrate trunk tissue [11].

Below we will outline a series of theoretical insights that can be used to fit gene expression data to decision structures (box 1). We will use the term *landscape* to refer to a dynamical system produced by parameters fixed at particular values and *landscape family* to refer to a parametrized set of dynamical systems in which changes to the parameters, which represent changes in extrinsic signals, change the systems dynamics.

Box 1. **A pseudo-algorithm for data fitting.**

These steps are described in more detail in the text.

1. ***From the experimental data identify the cell states (attractors) and transitions (unstable manifolds).*** Cluster and analyse cells, based on gene expression at suitable time points to determine attractors and transition states (§5). Do attractors have a well-defined correlation structure? Can transitioning cells be identified? What attractors are the cells transitioning between?
2. ***Hypothesize the decision landscape(s).*** Use the classification of landscapes (§4) and observations about cells states, attractors, transitions, and the way cells are allocated to attractors to identify the most probable landscape. Do the observed cell states and the transitions between them match any of the one- or two-parameter landscapes? Is decision-making binary and is it compatible with an all or nothing behaviour or a mixed production of downstream fates? Is the distribution of fates altered by modifying signalling/morphogens?
3. ***Construct a mathematical model to describe each of the hypothetical systems.*** Use the approach described in box 3 to build a dynamical model using a generalized gradient system and canonical examples from the elementary catastrophes.
4. ***Fit parameters to the most likely model(s).*** Choose an informative set of summary statistics to enable statistical model selection between the hypothesized models. Use an appropriate optimization algorithm to quantitatively fit parameters with the chosen summary statistics.
5. ***Validate/refute by prediction.*** Use the models resulting from the fit to design and to simulate uninvestigated experimental conditions. Confirm or contradict these predictions with new data.

## 3. Decisions and bifurcations

To link experimental data to mechanism, it is imperative to proceed via a model that describes the relevant underlying process, even if this does not contain a detailed molecular mechanism. In this section, we describe a mathematical picture of what happens in cellular decision-making. Later we will use this to provide a link to the data (box 1, §5).

In applied dynamical systems, the state of the system is usually given by an *n*-dimensional vector *x* = (*x*_1_, …, *x*_*n*_). The set of all *x* where we study the dynamics is called the *phase space*. Mathematical models of a GRN usually contain equations that describe the synthesis and interaction of the biological components (genes, RNAs, proteins, etc.) so that each *x*_*i*_ corresponds to the level of one of the relevant molecules. The resulting dynamical system describes how the levels of these species change over time. Although in typical GRN models, the *x*_*i*_ relate directly to physical entities, such as mRNA or protein molecules, this need not be the case. GRN models become infeasible and impractical when the number of physical entities considered is very large. We will discuss landscape models that have a minimal number of variables and parameters. These provide so-called *normal forms* and reproduce the important qualitative and quantitative dynamical features of the data. In such normal form models, the meaning of the *x*_*i*_ becomes more abstract but can usually be interpreted as a function of gene expression levels.

Gene expression in developing systems are generally subject to noisy fluctuations, but provided the noise is not too large we can view the system as a stochastic perturbation of a deterministic dynamical system (see §3.8). It is the deterministic part of the system that gives it the structural features that facilitate decision-making. Given an initial state *x*_0_ the deterministic dynamical system completely determines the future states *x*(*t*, *x*_0_) of the system as time *t* evolves. The corresponding path in phase space traversed as time advances is called the *trajectory* of *x*_0_.

It follows from the Waddington landscape picture, our understanding of molecular interactions, and models of GRNs that the relevant dynamical systems are dissipative in the sense that the stable states are either attracting rest points or attracting periodic orbits. We will focus here on those systems that do not have periodic orbits or more complex recurrent behaviour, such as chaos. Hence the key dynamical features of such a landscape are organized by rest points, i.e. states that do not move under the dynamics (such as attractors, saddles and repellors). Of course, there are interesting developmental systems that involve oscillations such as that regulating somitogenesis but here we restrict ourselves to systems where the decisions only involve rest points. We expect that much of our approach can be extended to the case where non-chaotic oscillations are present.

A dynamical system is called *structurally stable* if small changes to its parameters do not modify its qualitative structure. By contrast a *bifurcating* dynamical systems is one to which a small change alters the qualitative structure of the dynamics. There are a relatively simple set of conditions that are necessary and sufficient to determine if a system is structurally stable or bifurcating and also determine which bifurcating systems are likely to turn up in models. These were described by Smale [3,14], who radically developed earlier ideas of Morse [15]. The systems satisfying these conditions have become known as *Morse–Smale* (MS) systems. For systems, such as the ones we consider, in which the phase space is in *n*-dimensional Euclidean space the MS conditions also require a condition on the dynamics near infinity (see [2] electronic supplementary material, §I.1) that is satisfied for biological systems because trajectories must stay bounded and cannot escape to infinity because molecular numbers are limited.

The MS conditions are important because many useful results follow from them. Moreover, for any generic landscape family that only involves rest points and a finite number of periodic orbits, for all parameters that are not on the bifurcation set, the corresponding dynamical system is MS.

In order to introduce the key ideas, we discuss them in the context of systems that live in a 2D space. However, all these ideas carry over to an arbitrary number of dimensions as summarized in §3.7. More mathematical details can be found in [2]. In a 2D MS system the saddles will always be positioned at the intersection between a one-dimensional (1D) stable manifold (a ridge that separates basins) and a 1D unstable manifold (figure 2*b*). These 1D unstable manifolds play a particularly important role in decision-making because, as we explain below, they define the escape routes along which cell state transitions take place.

### 3.1. Decisions

Developmental decisions involve cells transitioning between fates by leaving the vicinity of attractor *A*, escaping from its basin, moving into the basin of another attractor *B* and transitioning to its vicinity. This generally happens when a change in signalling modifies the dynamical system resulting in either *A* disappearing in a bifurcation or the attractor *A* being so close to the basin of *B* that the cells can escape by a stochastic fluctuation, in which case the system is close to a bifurcation. Mathematically, we model the change in signals by regarding the parameters of any model as being functions of the signals, so that changing signals changes the parameters and this will alter the model and can cause bifurcations.

Thus, bifurcations play a key role in cell state transitions and cell fate decisions happen when parameters cross the so-called *bifurcation set*. As explained below, the bifurcation set divides parameter space into regions of qualitatively equivalent structurally stable landscapes (in figure 3*b* there are two regions, in figure 3*d* there are three regions).

The notion that bifurcations are involved in cellular decisions is relatively well accepted [16–23]. They have been postulated in a range of decision-making systems including the triggering of human promyelocytic HL60 cells to neutrophil differentiation [17], differentiation of progenitor FDCP-mix cells into either the erythroid/megakaryocyte or the myelomonocyte lineage [18], early mouse embryonic development [12,20,24], differentiation of a primitive streak-like cell population into mesodermal and endodermal lineages [21], somitogenesis [22] and the transition of haematopoietic stem cells to neutrophils [23]. However, current discussions are largely restricted to local bifurcations where saddles and attractors collide. Less consideration has been given to global bifurcations that alter the decision topology. However, these more complex bifurcations can result in landscape families that allow for more complex decisions (figures 7–12). Below we introduce different types of bifurcations that play a role in cellular decision-making.

### 3.2. The saddle-node or fold bifurcation

The typical bifurcation that destabilizes an attractor, as in the above examples, and allows escape from the attractor is known as a *saddle-node *or* fold bifurcation*. A change in parameters results in either an attractor colliding with a saddle and both disappearing or the inverse process occurring. It is particularly relevant because in a generic two-parameter family all other local bifurcations only happen at isolated points in the parameter space, whereas fold bifurcations occur on curves (called *fold curves*) that correspond to critical parameter values. An analogous result holds when there are more parameters. Thus, a fold bifurcation is the only type of local bifurcation that is expected to be observed. The bifurcations that occur at isolated points act as organizing centres for the fold bifurcations, as we explain below (§4).

### 3.3. Escape routes are determined by the unstable manifolds of index 1 saddles

**Figure 4.**
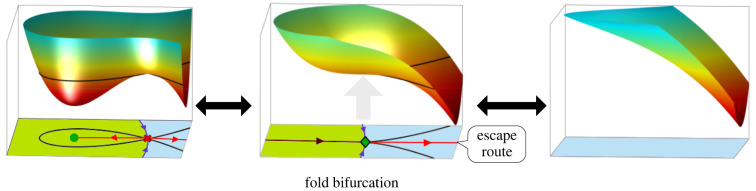
Fold bifurcation. Representation of a landscape before (left) a fold bifurcation, at the bifurcation point (middle) and beyond it (right). The attractor and saddle collide and disappear (middle) so cells in that basin of attraction follow the escape route (red) to another attractor.

At a fold bifurcation (figure 4), cells are forced to transition out of the now vanished attractor and into the adjacent attractor. As the parameters approach the fold curve (the bifurcation), the branch of the unstable manifold connecting the saddle to the adjacent attractor approaches what we term the *escape route*. As the parameters cross the fold curve the flow of the cells leaving the disappeared attractor are directed along the escape route into the newly available adjacent attractor. Of course, in saying this we are ignoring stochastic effects which could cause some cells to escape elsewhere.

Cell state transitions can also be caused by random fluctuations in the position of the cell state in the landscape. These can result in a cell spontaneously jumping over a stable manifold and moving from one basin to another (figure 3*c*). This is most likely if the starting attractor has a shallow basin of attraction and is near a saddle—hence the system is close to undergoing a fold bifurcation. In this situation, a relatively small fluctuation in a cell’s position in the landscape, caused by stochastic fluctuations in gene expression, might be sufficient to push it over the stable manifold and into the basin of the adjacent attractor. Such a transition is most likely to occur close to the saddle point as this represents the lowest point on the stable manifold. For a discussion about this and the relationship between the height of a saddle above an attractor and the expected time to escape the basin see, for example, [25,26]. Having escaped over the saddle, the cell state will follow the unstable manifold downhill to the new cell fate.

Thus whatever the cause, bifurcation or stochastic escape, the transition route to the new attractor will be determined by the unstable manifold of the relevant saddle.

### 3.4. Global bifurcations

Apart from a fold bifurcation, which changes the decision topology because a saddle and attractor disappear or appear, there is only one other generic way that altering a single parameter changes the decision structure. This is via a flip in an unstable manifold (as in the binary flip landscape §2, figure 3). In this case, as the critical parameter value is approached, the unstable manifold of a saddle *S*, which connects to attractor *A*, approaches the stable manifold of another saddle *S*′ (shaded panel in figure 3*c*). At the critical value it loses connection to *A* and forms a *heteroclinic connection* to *S*′. In a heteroclinic connection, the flow of the system goes from the saddle *S* towards the second saddle *S*′. Thus, we call *S* the *source* saddle and *S*′ the *sink* saddle. This arrangement is not structurally stable: any small perturbation breaks the connection. Beyond the critical value, the unstable manifold makes a new connection to attractor *B*. We call this a *flip bifurcation*.

Whereas a fold bifurcation is local in that it only affects the dynamics in a small region around the bifurcating rest point, the flip bifurcation is global in that the resulting reconfiguration of the landscape changes the global structure of the dynamics because the unstable manifold now goes to a completely different attractor. The flip is therefore known as a *global bifurcation*. For such a flip bifurcation to occur the phase space needs to be at least 2D. Importantly, for decision-making, the effect of a flip bifurcation is only observed when, after the flip has happened, either the state stochastically escapes the attractor over the saddle associated with the flipped unstable manifold or when this saddle is destroyed in a fold bifurcation with the attractor representing the progenitor state (or close to bifurcating). This will happen, for example, when a changing signal weakens the attraction and causes the bifurcation.

### 3.5. Decision topology

A graph *G* can be constructed that describes all the possible transitions in a generic landscape family and for systems with not too many attractors we can list all possible decision topologies. This uses the fact that the escape route of a cell transitioning from one state to another is determined by the unstable manifold of the saddle associated with the initial attractor and the destination attractor. It applies both to transitions resulting from a bifurcation and those caused by a stochastic fluctuation. It does not depend upon the state space being 2D and applies equally in higher dimensions (see §3.7). In these graphs, the nodes correspond to the attractors and an edge connects two nodes if there is a saddle that has a one-dimensional (1D) unstable manifold that connects the corresponding attractors.

In all of the cases discussed above, the escape route of a cell transitioning from one state to another is determined by the unstable manifold of the saddle associated with the initial attractor and the destination attractor. Hence, even when a decision is initiated by a stochastic fluctuation, the deterministic structure of the dynamical system determines the decision outcome. This means that, given an MS system, a graph can be constructed that describes all possible transitions in a system. In this graph, the nodes correspond to the attractors and an edge connects two nodes if there is a saddle that has a 1D unstable manifold that connects the corresponding attractors. In higher dimensions, we have a similar situation (see §3.7).

**Figure 5.**
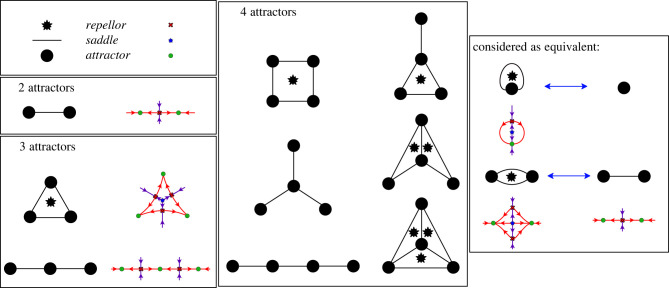
Decision structures with up to four attractors. Nodes correspond to attractors and edges to saddles with unstable manifolds that connect the corresponding attractors. The asterisks denote the existence of at least one saddle of index 2. In 2D systems such a saddle is a repellor.

To list all possible decision topologies, we first simplify things by noting that if *A* and *B* are attractors, there can be multiple connections between them but that all these connections correspond to the same decision, i.e. a transition from *A* to *B* or vice-versa. We delete these repeats in the graph, keeping just one. We also delete all connections where *A* = *B*. The resulting graph is called the *decision graph* and its topology the *decision topology* because it lists all possible transitions in the system. In figure 5, we show all decision topologies for MS systems with two, three and four attractors.

### 3.6. The landscape potential, alone, does not determine cell state transitions

For MS systems with only rest points and no periodic orbits or other complex recurrent behaviour such as chaos there is always a *Liapunov* or *potential function* [14]. This is a function that is stationary at rest points and decreases along trajectories that are not periodic [27]. Thus, the trajectories flow downhill with respect to this function.

**Figure 6.**
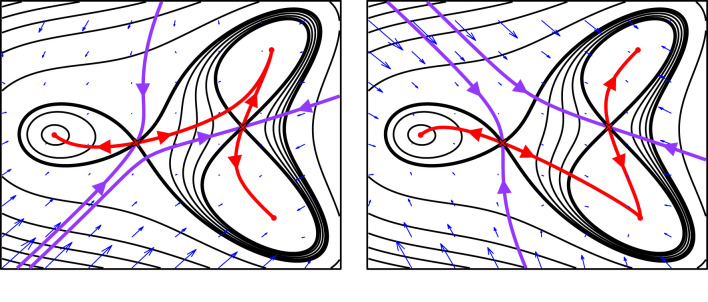
The potential alone does not determine the dynamics. Two qualitatively different dynamical systems that have exactly the same landscape potential. The potential, alone, does not determine the position of the unstable manifolds of the saddles and therefore does not determine to which attractor transitioning cells go. In a gradient system using the standard Riemannian metric (see box 2) the trajectories are always perpendicular to the contours but this does not have to be the case. Dynamical systems that flow downhill, from higher altitude to lower, can cross the contours at angles other than $90^{\circ }$ (see box 2). In this example, we show a modification that produces a flip in the unstable manifold of the saddle. This example shows that the potential does not determine the decision structure.

The existence of a potential function provides the link between dynamical systems theory and the Waddington landscape view of differentiation and it offers an intuitive way of picturing the dynamics. There is however an important qualification to make, because, in all but the simplest MS systems, a potential is not sufficient to determine the dynamics. Indeed, as demonstrated in figure 6 the potential function does not necessarily even determine the qualitative form of the dynamics or the possible developmental transitions. Additional information is required to describe accurately the dynamics and determine the decision structures (figure 6 and box 2).

Box 2. **Gradient systems.**

The general idea of a gradient system involves both a potential *F* and a Riemannian metric *G*. Such a Riemannian metric provides at each point *x* = (*x*_1_, …*x*_*n*_) of the phase space an *n* × *n* symmetric positive definite matrix *G*(*x*) = (*g*_*ij*_(*x*)). Then the dynamics are given by the differential equation

3.1 $$\dot{x}=-G^{-1}\nabla F,$$

where ∇F is the vector of partial derivatives of *F*. Note that *F* is a potential function for this.

In some discussions, it is assumed that *G* is the identity matrix so that the Riemannian metric is the standard one but we always use the more general meaning.

It is important to note that changing *G* does not change the rest points and does not change the topological type of the rest points in that the dimension of their stable and unstable manifolds is unchanged.

However, as we show in figure 6 such a change can alter where the unstable manifolds go. The examples in figure 6 correspond to the same potential

$$F(x,y)=x^{3}-2xy^{2}-0.4x^{2}-3x-y+\frac{x^{4}+y^{4}}{4}$$

and different constant matrices *G* for each panel as follows:

$$G=\left(\begin{array}{cc} 1 & -0.6\\ -0.6 & 1 \end{array}\right)\;\mathrm{and}\;G=\left(\begin{array}{cc} 1 & 0.6\\ 0.6 & 1 \end{array}\right).$$

Changing the metric flipped the unstable manifolds.

A consequence of this is that it is important to consider the dynamical system and not just the potential function. The dynamical system contains extra information that is needed to describe the dynamical behaviour. This means that to construct a model from data it is necessary to fit the dynamical model and not simply the potential. Defining a potential is insufficient to determine the dynamics. For model building, a practical approach is to use a *Riemannian metric* in combination with the potential function as explained in box 3.

Box 3. **Using gradient dynamics to model decision-making.**

Smale’s result indicates that every MS system has a potential [3]. Building on this idea, it can be deduced that all rest-point-only MS systems can be well represented by a gradient system [2]. In turn, it follows that gradient systems are an effective tool to model decision-making systems. However, the result is somewhat sophisticated and requires careful discussion. By a gradient model we mean one as in box 2 involving both a potential and a Riemannian metric. The local dynamics around a rest point in a gradient system has a special structure because the Jacobian at these points is a symmetric matrix. For example, in a general MS system, trajectories can spiral into an attractor or saddle, whereas this is forbidden in a gradient system. Fortunately, it is possible to correct for this without changing the global dynamics substantially. Suppose that we have a general MS system with only fixed points. Let *F* be any smooth potential for this system. Choose a small disc *U*_*i*_ around each of the rest points *β*_*i*_. These can be as small as you like. One can show (see [2] electronic supplementary material, appendix A, [28]) that you can modify the system inside the *U*_*i*_ so that the resulting system is a gradient system defined by the potential *F* and some Riemannian metric. Moreover, it can be chosen so that outside the *U*_*i*_ all the stable and unstable manifolds of the rest points are changed from those of the original MS system by no more than an arbitrary small amount that can be prescribed in advance. Importantly, the corresponding connections between attractors are preserved. The perturbation inside the *U*_*i*_ might be large but the type of rest point (e.g. attractor or saddle) is preserved. In particular, the decision topology is maintained and the escape routes of the original and gradient systems are as close as you want.

This result indicates that if you want to postulate a model to represent a GRN with no periodic behaviour you might as well use a gradient model. On the other hand, you must be prepared to allow for a Riemannian metric that is different from the standard one (where the matrix *G* in box 2 is the identity matrix).

This approach to constructing quantitative parametrized models is further facilitated by using the canonical potentials given by catastrophe theory [4,5,7]. Components of a model can be combined by a process of gluing canonical potentials together to obtain a full model as carried out in [10,11]. These can be parametrized in a universal way by using the unfolding parameters given by catastrophe theory [5]. For example, the family in figure 12*b* corresponds to

$$F(x,y;a,b)=x^{3}-2xy^{2}-0.4x^{2}+ax+by+\frac{x^{4}+y^{4}}{4}.$$

This is essentially the elliptic umbilic from Thom’s list of elementary catastrophes [4] with a term *x*^4^ + *y*^4^ added to make it compact, i.e. to make it have the right behaviour at infinity in the phase space.

### 3.7. Higher dimensional systems

For simplicity, we have focused on systems with a 2D phase space. However, all the results we discuss are valid in the *n*-dimensional case, with appropriate modifications [2]. Beyond 2D, saddles can have higher dimensional stable and unstable manifolds. However, in order to study decisions, the saddles with multidimensional unstable manifolds are generically irrelevant in that they are not involved in transitions from bifurcations or stochastic escape from attractor basins. We only have to consider the saddles with 1D ones (so-called *index 1 saddles*) and the connections they form between attractors.

There is an important caveat to these statements. Although in a generic MS system the 1D unstable manifolds of index 1 saddles will always miss higher index saddles (i.e. they do not converge to a higher index saddle but miss it and go to an attractor), this is not the case for some systems if multiple identical cells are involved. Such systems have a symmetry (interchanging cells) and are therefore not generic and in this case a 1D unstable manifold can be captured by a higher index saddle. Of course, in reality cells will not be identical and then the 1D unstable manifold will miss the higher index saddle. However, it may still pass close to it and in this case the higher index saddle may play an important role in the dynamics of the system. For example, because of the symmetry there is likely to be many attractors and the higher index saddle may determine which of these the 1D unstable manifold captures. This is the case for a recent model for sensory bristle patterns in a fly system [29] and for a model describing the allocation of cell fates between epiblast and primitive endoderm lineages in mouse embryonic stem cell cultures in [24].

Nevertheless, the theory of generic local bifurcations of codimension one and two (i.e. those we are concerned with) is agnostic to the dimension of the phase space as is the flip bifurcation. Moreover, in the situations considered here the decision structures for systems with three and four attractors in *n* dimensions agree with those in 2D [2].

### 3.8. The link to stochastic systems

Stochasticity resulting either from intrinsic fluctuations, which arise because of the limited number of molecules in a cell, or extrinsic fluctuations, produced by the highly dynamic and inhomogeneous cellular environment of the cell, affect cell decision-making (e.g. [18,30–37]). How does this link to our discussion? Perhaps the most sophisticated models of cellular differentiation that take stochasticity into account are those that use stochastic perturbations of dynamical systems (e.g. [25]) or Markovian jump processes (e.g. [38–40]). These usually contain a parameter Ω which represents the system size. This is related to the number of particles in the system.

Under reasonable conditions such models give rise to a specific potential, often called the quasi-potential. Moreover as Ω grows these systems converge to a deterministic dynamical system of the sort we have been discussing for which the quasi-potential is a Liapunov function. The key properties of this quasi-potential concern its relation to the stationary distribution $\mu^{\Omega }$ of the system and the asymptotics of average times for a stochastic trajectory to escape an attractor basin. The distribution $\mu^{\Omega }$ gives the probability of finding a trajectory in any part of the phase space for large times and tells us where these are concentrated as time advances. Both of these features are related to the depth of the basin in the quasi-potential [25]. As Ω increases, trajectories will be increasingly concentrated around the attractors and they will take exponentially longer to escape from any basin. These changes are described by the quasi-potential.

When Ω is sufficiently large, which should be the case for most biologically relevant systems, most noise-driven escapes from a basin of attraction happen near an index 1 saddle [25]. The uphill part of the route to the escape, near the saddle point, may be non-trivial (e.g. [36,41]) and can be found by minimizing a certain action [25,32,37,41,42]. However, when it gets over the saddle the dominant route is then down the unstable manifold of the saddle. This links nicely to the approach we have described as the noise structure of the Markov jump process will naturally determine an ordering of the heights of the saddles of the deterministic system [25] and this determines the qualitative structure of the quasi-potential.

In addition to the relationship of the quasi-potential to single transitions from one basin of attraction to another, there is a process on a longer timescale describing the long-term statistics of transitions between the different attractors that is described by a global landscape obtained by gluing local quasi-potentials together [25,26].

## 4. Characterizing two-parameter landscape families

In the previous section, we presented the mathematical picture corresponding to a landscape and the most relevant bifurcations that can happen. In this section, we discuss how these elements define landscape families that depend on a set of parameters. We will focus on the situations with one or two parameters. Each parameter corresponds to a signal (or a combination of signals). In order to model how these signals affect decision-making it is key to organize and classify the possible families so one can resort to this dictionary while building the model.

As mentioned above, when the parameter space is 2D, fold and flip bifurcations occur on smooth curves in parameter space. These *fold* and *flip curves*, respectively, constitute the bifurcation set. They divide the parameter space into regions of qualitatively equivalent landscapes and describe the changes in dynamics that happen as the bifurcation set is crossed. In addition, there are points where pairs of curves meet. At these points (often called *codimension two points*) more complex bifurcations occur. These points provide a natural way to characterize landscape families because they define the ‘corners’ of regions corresponding to qualitatively equivalent landscapes and the families around them represent interesting topologies. In the following sections, we describe specific examples of two-parameter landscape families and show how they can be understood by considering codimension two bifurcation points.

### 4.1. Bistability: the standard cusp

**Figure 7.**
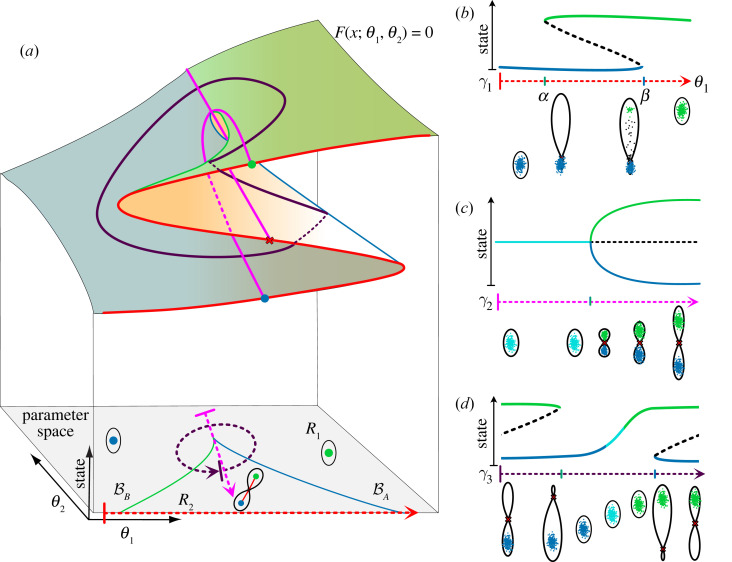
Bistability: the standard cusp. (*a*) Catastrophe manifold and bifurcation set. In 2D parameter space, the blue and green lines represent the bifurcation set with the colour of the line corresponding to the attractor involved in the fold bifurcation that occurs on that fold line. The dashed arrows indicate example paths in parameter space, corresponding to a continuous change in an extrinsic signal. The 2D catastrophe manifold on top of the parameter space shows the fold lines and the preimages of the different paths that are sketched with more detail in panels (*b*,*c*,*d*). In the region *R*_2_ (inside the cusp, with three sheets above), there are two attractors *A* and *B* separated by a saddle *S*. When the parameters move out of *R*_2_ to *R*_1_ (outside the cusp, with one sheet above) by crossing $B_{A}$ (respectively, $B_{B}$) the attractor *A* (respectively, *B*) and the saddle undergo a fold bifurcation which destroys *A* (respectively, *B*). Paths *γ*_1_ across the cusp, *γ*_2_ vertically through the cusp, and *γ*_3_ around the cusp correspond to the hysteresis, pitchfork and cusp smooth swap diagrams (respectively). (*b*) Bifurcation diagram of a genetic switch. Change in the rest points of a bistable system, as a parameter *θ_1_* changes. A single attractor (green) is present when *θ* = 0. When *θ_1_* crosses the bifurcation point (*α*), a saddle (dashed curve) and a new attractor (blue) appear. When the parameter crosses a second bifurcation point (*β*) the initial attractor (green) collides with the saddle and disappear causing the cells in it to transition to the available attractor (blue). The coexistence of both attractors between *α* and *β* results in bistability and hysteresis. (*c*) *Pitchfork.* As a parameter changes along *γ*_2_, an initial attractor becomes a saddle point and two attractors appear at each side. The system is symmetric with respect to the saddle point. (*d*) *Smooth state swap.* The path *γ*_3_ around the cusp shows how cells transition from *A* into *B* without a step-like switch. When the path crosses the bifurcation set, the bifurcating attractor is empty and hence no cells are forced to transition, instead, going around the cusp in *R*_1_ changes the gene expression in a smooth continuous way.

Bistable systems are common in biology and have been used, for example, to describe genetic toggle switches [43]. These are often studied by varying a signal (parameter) and noting the characteristic *S-* or *Z*-shaped bifurcation curve (figure 7*b*). A typical genetic toggle switch exhibits hysteresis and has two fold bifurcations (*α* and *β* figure 7*b*) where the system switches between mono- and bi-stable regimes.

If there are two parameters, then fold bifurcations, like those at *α* and *β* in figure 7*b*, occur on fold curves and these can meet at a cusp point. This defines the *standard cusp family* (figure 7*a*) [5,7]. Cusps are relatively common and there are simple criteria that indicate their presence in a system [2]. In this family, the bifurcation set consists of two fold curves ($B_{A}$ and $B_{B}$) corresponding to the two different attractors that meet at a point C where there is a cusp (figure 7*a*). The curve defined by the bifurcation set separates the parameter space into two regions *R*_1_ and *R*_2_. In *R*_2_ there is bistability, that is, a pair of attractors *A* and *B* connected by a saddle *S*. Varying the parameters so as to cross $B_{A}$ (respectively, $B_{B}$) results in a fold bifurcation that destroys *A* (respectively, *B*).

A bistable bifurcation produced by varying a signal can be viewed as a path in the 2D parameter space of the standard cusp family. Examples are shown as *γ*_1_, *γ*_2_ and *γ*_3_ in figure 7*a* and the cusp is the non-trivial generic way these are organized. In figure 7, we identify paths that give the *S* (or *Z*) shaped bifurcation diagram that produces hysteresis (*γ*_1_, figure 7*b*), the pitchfork where one state splits into two new states (*γ*_2_, figure 7*c*) and a smooth swap path that allows cells to change state without a step-like transition (*γ*_3_, figure 7*d*). From these we see that the pitchfork is non-generic because it requires the parameter to pass through the cusp point exactly. Any small perturbation would cause the path to miss it. The smooth swap path (figure 7*d*), on the other hand, raises the interesting possibility that topologically equivalent genetic toggle switches can produce either abrupt or smooth transitions in cell states depending on the details of the parameters and the effect of signals.

### 4.2. The choice landscape: the dual cusp

**Figure 8.**
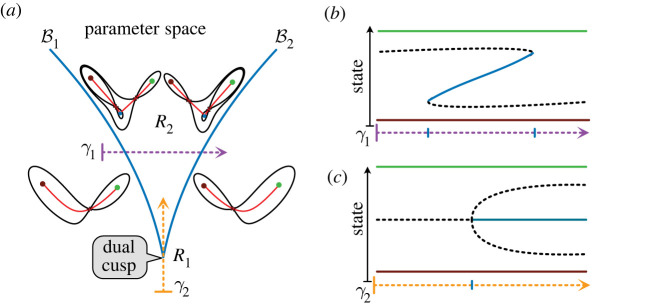
The choice landscape: the dual cusp. (*a*) Bifurcation set. The solid lines show the bifurcation set. The dashed arrow shows a path in parameter space. In the region *R*_2_ (inside the cusp, above), there are three attractors separated by two saddles. When the parameters move out of *R*_2_ to *R*_1_ (outside the cusp, below) by crossing $B_{1}$ (respectively, $B_{2}$) the central attractor (blue) and the right (respectively, left) saddle undergo a fold bifurcation which destroys the attractor. (*b*) The path (*γ*_1_) indicated gives a 1D representation of the choice family and the available transitions from the central attractor. The duality between the hysteresis diagram and the middle part of this diagram is apparent. (*c*) The path (*γ*_2_) indicated shows a pitchfork bifurcation where the central saddle becomes two saddles and a new attractor. The duality between this diagram and figure 7*c* is apparent.

The choice landscape (figures 3*b* and 8) is produced by a family of landscapes with bifurcation sets that also comprise two fold curves meeting at a cusp point. However, in this family of landscapes each curve corresponds to the fold bifurcation of the central attractor with one of the two saddle points. The point where the two curves meet is a *dual cusp*. Thus, while the standard cusp describes the addition (removal) of an attractor and a saddle to an existing attractor, the dual cusp involves the addition (removal) of an attractor and a saddle to an existing saddle. In a system with no trajectories going to infinity, which is the case for the systems we consider, peripheral attractors are also present. The consequence of this is that the inside of the dual cusp is a tristable region and the outside is bistable (figure 8*a*).

### 4.3. Fold crossing points: a French flag

**Figure 9.**
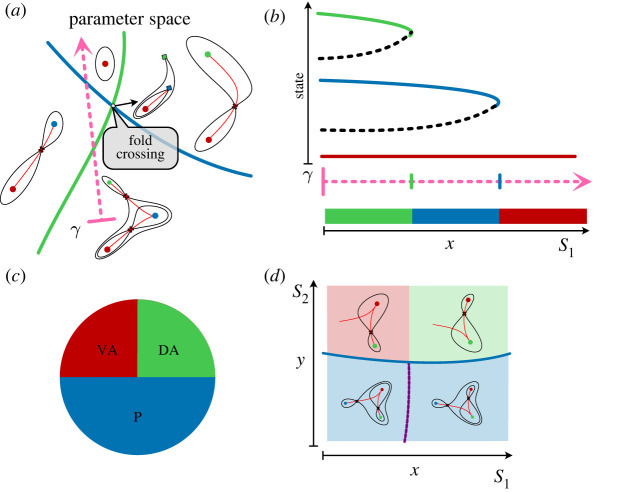
Fold crossing points of bifurcation curves. (*a*) Bifurcation set. Landscapes around a fold crossing, corresponding to two adjacent attractors, one central (blue), the others peripheral (green, red). The solid lines represent the bifurcation set with the colour of the line corresponding to the bifurcating attractor. (*b*) *French flag model.* The path *γ* in the 2D parameter space in (*a*) produces the bifurcation diagram shown in (*b*). Suppose that the cells are spatially organized in 1D with coordinate *x* and all cells start in the green attractor. Cells are exposed to a signal *S*(*x*) which increases monotonically with *x*. If *x* is small so that the signal is small, cells will stay in the green attractor. For larger values of *x* which will have higher values of the signal, the cells will transition to the blue or red attractor. Moreover, cells will stay in the attractor when the signal decreases. Since the signal is a monotonic function of *x* this results in a French flag pattern. (*c*,*d*) Meinhardt boundary model. When there are two signals, a landscape with a flip curve endpoint as in (*d*) can produce patterning in a similar fashion. In such an arrangement, a system transitions between three states in response to a combination of two signals. In this model the physical space of the tissue is 2D: one dimension, *x*, has a gradient of signal *S*_1_ and the other, *y*, has a graded signal *S*_2_. All cells are assumed to start in the blue attractor. If signal *S_2_* is small enough that the point (*x*, *y*) is below the blue fold curve of the bifurcation set, then the cell stays in this attractor. If (*x*, *y*) is above the fold curve this attractor is destroyed in a fold bifurcation and the cells transition to one of the other attractors. Because of the flip, when (*x*, *y*) is in the red region (respectively, green region) the state transition to the red (respectively, green) attractor. Moreover, all cells stay in the respective attractor when the signal *S*_2_ decreases. Consequently, after a pulse of the signals the spatial patterning is as shown in (*c*) with three distinct regions of tissue.

**Figure 10.**
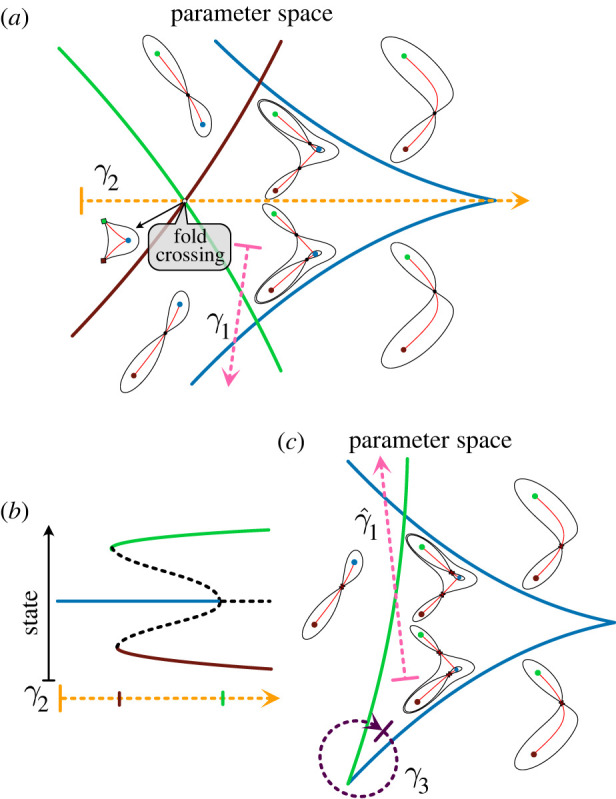
Families with a dual cusp. (*a*,*c*) Bifurcation sets. Two landscape families with a dual cusp. The solid lines show the bifurcation set with the colour of the line corresponding to the attractor involved in the fold bifurcation. The dashed arrows illustrate paths in parameter space. (*a*) The region where there are three attractors is bounded by a dual cusp and fold curves with crossings. The crossings that can occur are highly constrained [2]. Path *γ*_1_ corresponds to a French flag landscape. (*b*) *Symmetry breaking.* This bifurcation diagram corresponds to the path *γ*_2_ in (*a*). It is the dual cusp version of the pitchfork bifurcation that occurs in the standard cusp. Like the pitchfork, it is not generic but in the exceptional case where there is a $Z_{2}$-symmetry such as when the system describes the behaviour of two identical cells it becomes generic and describes an interesting symmetry breaking bifurcation (see [24] for an example). (*c*) A three attractor region bounded by a dual and standard cusp. Path $\hat{\gamma_{1}}$ corresponds to the French flag landscape. Path *γ*_3_ corresponds to the cusp smooth state swap of the green and blue attractors.

**Figure 11.**
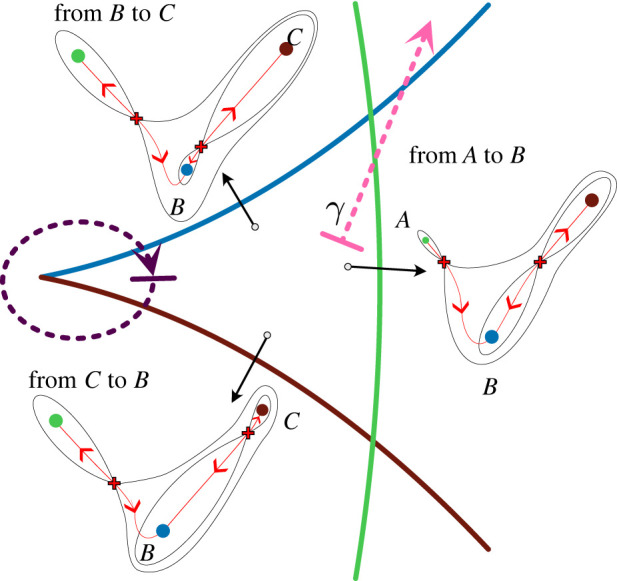
Families with one standard cusp and no other cusps. The landscape family with the solid lines representing the bifurcation set. The colour of the line corresponds to the attractor involved in the fold bifurcation. Path *γ* corresponds to a French flag landscape. A cusp smooth state swap path is also sketched.

Instead of meeting at cusps, fold curves can also cross one another. These represent points in parameter space where there are two independent fold bifurcations (figures 9*a* and 10*a*). Fold crossings have several implications for the structure and behaviour of a landscape family. First, only certain crossings can occur when they affect connected attractors and some crossings introduce topological constraints that imply the presence of cusps or dual cusps [2]. For example, the dual cusp in figure 10*a* must be present because the fold curve of the central attractor (blue) crosses the curves of the adjacent two attractors (green and brown curves). Moreover, the presence of the three crossings of the three outer curves in figure 11*b* implies that there must be a complex bifurcation set within them (blue deltoid) (see [2] fig. 4 and electronic supplementary material, §§4–6). Hence the identification of a specific fold crossing in a system can be used to deduce additional features of a landscape family.

Second, if the two fold curves that cross correspond to adjacent attractors in a (at least) three attractor family, a model for a ‘French flag’ pattern results (figure 9*b*). In such an arrangement, a system transitions between three states in response to a smooth change in a parameter (signal). Moreover, such a landscape results in a system that is multistable. Hence cell identity, once established, is predicted to be maintained after withdrawal of the patterning signalling. This contrasts with monostable systems which would be dependent on continued signal provision to maintain identities. Experimentally testing the effect of signal removal would therefore distinguish between these possibilities.

### 4.4. The flip landscape: termination of flip curves

**Figure 12.**
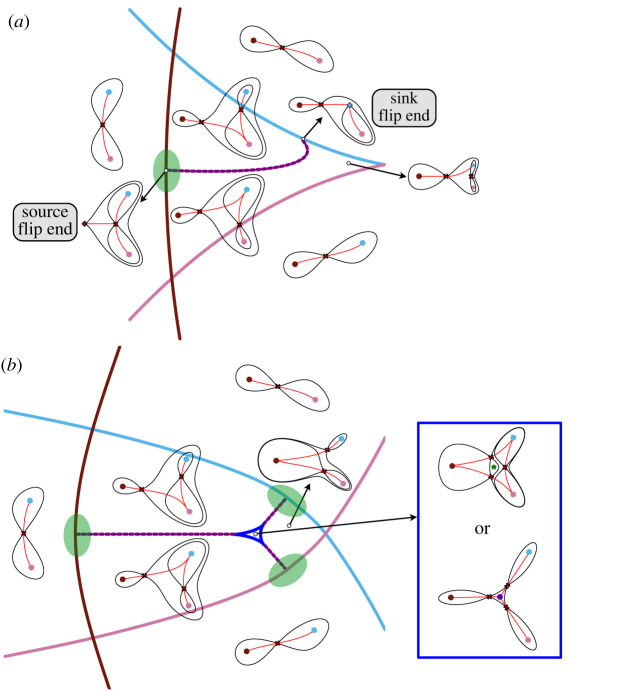
Families with flip curves. (*a*,*b*) Bifurcation sets. The bifurcation sets for two landscape families: the solid lines indicate fold curves and the dashed lines are flip curves. The colour of the fold line corresponds to the attractor involved in the fold bifurcation. Examples of landscapes in the family are sketched. Both families contain the flip landscape in the areas marked in green. (*a*) One flip with a cusp. The two attractors connected to the sink saddle can undergo a cusp bifurcation. (*b*) With no cusps. This family contain three instances of the flip landscape. The region enclosed by the blue deltoid corresponds to landscapes with an extra pair of critical points: a saddle and either a repellor or an attractor (as shown in the blue box). Generically, the flip curves enter the deltoid avoiding the cusps and terminate in one of the three smooth edges of the deltoid. This family is part of the compactified elliptic umbillic catastrophe.

The flip landscape (figure 3*d*) is produced by a family of landscapes in which the bifurcation set comprises a flip curve that terminates on a fold curve. The fold curve represents the bifurcation involving the top saddle and the progenitor state attractor. A flip curve ends when it meets a fold curve and one of the two saddles involved in the heteroclinic connection is destroyed by a fold bifurcation. If the bifurcating saddle is associated with the source attractor, then the two curves meet transversally. Otherwise they meet tangentially (figure 12*a*). We call such points *source and sink flip ends*.

The family of landscapes around the source end of a flip curve can allow the proportional allocation of cells to two fates (figure 3). It can also be used to produce a pattern similar to that of the Meinhardt boundary model [44,45] (figure 9*c*,*d*). Meinhardt suggested this model as a mechanism by which cells in an imaginal disc could measure circumferential location and polarity by comparing two signals (figure 9*c*).

### 4.5. Characterizing three attractor landscape families

Landscape families containing three attractors are of particular significance because of the prevalence of decisions involving three states: a progenitor choosing between one of two committed states. We described two such landscapes in §2. Here we consider what else can occur and how these are related to bifurcations. An understanding of how bifurcation sets for three attractor families relate landscapes to one another provides the basis for a classification scheme of decision-making.

A series of rules dictate how the different components of a bifurcation set (fold and flip curves, cusps, dual cusps, flip curve terminations and fold crossing points) fit together in a parametrized landscape family [2]. Using these rules, we can build a list for the simplest landscape families with three attractors and no trajectories going to infinity.

1. *With a dual cusp and no other cusps: *figure 10*a.* The open-ended curves can extend to the boundary of parameter space or end in standard cusps (as is the case in the butterfly catastrophe [4,7]). This landscape family contains the binary choice landscape, paths that give the bifurcations observed in French flag patterning and a path corresponding to the pitchfork bifurcation.
2. *With a dual cusp and a standard cusp*: figure 10*c*. Similar to the previous landscape family, it contains the binary choice landscape and paths through this also give the French flag patterning. In addition, it contains paths that allow the cusp smooth state swap (see §4.1).
3. *With a standard cusp and no other cusps: *figure 11*a.* In this case, the possible transitions are highly restricted. State *A*, which is not involved in the cusp, can only bifurcate towards the adjacent state *B*. States *B* and *C*, which are involved in the cusp, can exchange cells in both directions depending on parameter values. A cusp smooth state swap between *C* and *B* is also possible.
4. *With a standard cusp and a flip curve: *figure 12*a.* In this case, a flip curve terminates at a source and a sink end. It contains the binary flip landscape. This is the landscape used in [10] to model the early development of the vulva in the worm *C. elegans*. It also contains a subfamily that gives the Meinhardt boundary model.
5. *With flip curves and an outer boundary of three fold curves with no cusps: *figure 12*b.* In this family of landscapes, a set of bifurcation curves that make a deltoid inside the three attractor region implies two additional critical points: a saddle and either a repellor or an attractor (figure 12*b*). This also contains the binary flip landscape and is equivalent to the landscape family used by Corson & Siggia [8,9].

Importantly, families 1, 2 and 3 correspond to essentially 1D dynamical systems because the three attractors are ordered in a specific way that does not change on a smooth 1D invariant manifold. This places a constraint on the allowable transitions between cell states and the order in which these can take place.

### 4.6. Beyond three attractor families

The more attractors a system contains, the more distinct possible arrangements between attractors and unstable manifolds. For four attractors, there are six possible arrangements (figure 5). The case of four attractors organized around one repellor corresponds to the family defined by the double cusp catastrophe. This landscape naturally appears as the coupling of two bistable systems. A compactification of all elementary catastrophes lives inside the family defined by the double cusp catastrophe (a 7D family).

## 5. Beyond the metaphor: incorporating experimental data with dynamics

### 5.1. Critical transitions and correlation

To connect the theory to experimental observations requires ways to identify key landscape features from experimental data. How can data be used to recognize attractors, bifurcations and unstable manifolds? Signature gene expression patterns and clustering algorithms have been used to identify attractors and end-states corresponding to specific cell types [11,12,20,21,35,46–51]. In addition, much interest has focused on identifying cells at decision points and understanding the detailed structure of transitions at or near a bifurcation [23,35,52]. The aim of these methods is to determine the critical points where cells change state and provide criteria that identify these from experimental data.

Comparing correlations in gene expression between cells has been used to define signatures of bifurcations and attractors. Specifically, if *g*_*ij*_ is the expression level of gene *j* in cell *i*, the *gene–gene correlations* *γ*_*jj*′_ are defined as the Pearson correlations between *g*_*ij*_ and *g*_*ij*′_ averaged over the relevant cells. By contrast, for a given set of genes, the *cell–cell correlation* is defined as the Pearson correlations between the vectors *c*_*i*_ = (*g*_*ij*_) averaged over all cell pairs. During a transition, the cell–cell correlation is predicted to be low but the absolute value of the gene–gene correlation relatively high [35]. The idea is that if the vectors *c*_*i*_ are in a line, then the cell–cell correlations are zero and the gene–gene correlations are all ±1. The low cell–cell correlation can be clearly pictured as cells spreading out along the low-dimensional unstable manifold of the bifurcating saddle to reach the newly available attractor. Conversely, the high gene–gene correlation is a result of cells being squeezed onto the unstable manifold by the transversal contraction of the flow. As cells then approach a new attractor the gene–gene correlations of the cells should adopt values characteristic of that attractor, reflecting the activating and repressing characteristics of the part of the GRN that is active at that attractor. Mojtahedi *et al.* [35] suggest that at attractors the absolute value of the gene–gene correlation is lower than at bifurcations while the cell–cell correlation is relative high. Nevertheless, in [11] (Methods electronic supplementary material, §2.3) the convergence to an attractor correlation structure given by *γ*_*jj*′_ is clearly observed and each attractor has a distinct correlation structure. Hence, whether low gene–gene correlations will be a reliable metric for identifying attractors is less clear and is likely to depend on the details of the attractors and the genes assayed.

Others have examined the effect on gene–gene correlations resulting from the critical slowing down that occurs as fold or pitchfork bifurcations are approached [23]. Further work will be necessary to define clearly transition states and decision points. This will help to devise and test the most appropriate methods for identifying cells at attractors and bifurcation points.

### 5.2. Dimension reduction and temporal ordering

Using data from a population of differentiating cells to determine the unstable manifold of a system could also provide a link between data and theory. For this, techniques that use single-cell transcriptome data to generate the so-called pseudo-temporal orderings or trajectories are promising. A key challenge in these methods is dimension reduction. Current approaches use manifold learning techniques and methods from graph theory to represent data as a neighbourhood graph embedded in two or three dimensions [53–56]. Trajectories are then inferred from these graphs (see [57] for a review). The nodes of the graphs correspond to cells and are connected if they are close in the higher-dimensional gene expression space. Because these edges cannot be clearly associated with dynamics and may be spurious and noise-related, it is hard to decide whether cells are in fact meaningfully connected or disconnected. Moreover, these methods often rely on ad hoc techniques that produce non-smooth mappings from high to low dimensions. They therefore appear less appropriate for deducing the dynamics. Nevertheless, RNA-velocity techniques [46,58] might reduce the spurious connections problem. Methods using partition-based graph abstraction, which localize such graphs, are another promising approach [59] to tackle these problems and might be combined with the approach described below (§5.3). Mass transport approaches are currently popular (e.g. [60]) and in the developmental context have been proposed as a way to introduce dynamics into expression data [50]. However, the link between such approaches and the dynamics of an underlying GRN is currently unclear.

### 5.3. Gene-free normal form models fit to data

To begin tackling the challenge of connecting theory with data, we and others [8–11] have used the idea of a normal form to investigate experimental data. A normal form represents a model with the minimum number of parameters necessary to reproduce all the quantitative and qualitative dynamical features of the data. In catastrophe theory and bifurcation theory, this notion can be defined rigorously (e.g. [7]). The strategy is to take advantage of the classification of simple systems described above (§4), to determine which has the most plausible qualitative correspondence to the experimental data and then use the normal form of the model (box 3) to fit the data. Currently, fitting relies on using summary statistics of the data that represent the proportions of cells associated with specific attractors of the system. An optimization approach, using stochastic simulation algorithms such as approximate Bayesian computation using Markov chain Monte Carlo (ABC MCMC, which uses comparison with simulated data instead of a likelihood to assess the goodness of fit of a certain parameter set) and particle-tracking algorithms [61,62] in which data are split into test and validation sets, is used to fit the parameters of the chosen normal form and ensure models are not overfit. The resulting state variables are not necessarily directly related to gene expression levels and are somewhat abstract but they may be seen as nonlinear functions of these expression levels. Nevertheless, the models make quantitative predictions about proportions of specific cell fates that are testable with new experiments.

This strategy has been taken to investigate the vulva development of the worm *C. elegans* [8–10]. In this system, six precursor cells with equal developmental potential develop into three different fates depending on the signals (a combination of EGF and Notch). The statistics of final fate decision were used to analyse this system. Corson & Siggia [8] constructed a model using the insight that a specific configuration of three attractors that bifurcate appropriately is needed. By using an essentially linear model with a nonlinear wrapper, they were able to minimize the number of parameters and focus on the essential ones. This enabled the fitting of a large number of experimental observations and the finding of a number of interesting new experimental predictions. The full geometry of this model is equivalent to the compactified elliptic umbilic family (figure 12*b*). Using a geometric model based on catastrophe theory, a simpler landscape family (figure 12*a*) was demonstrated to be sufficient to represent key characteristics of the vulval system [10]. A framework using an ABC method based on sequential Monte Carlo sampling [61] was developed to fit the parameters of the landscape and the effect of the different signals on them.

The framework developed in these papers was extended to build a landscape model describing the *in vitro* differentiation of mouse embryonic stem cells into neural or mesodermal lineages in response to WNT and FGF signalling [11]. Single-cell (FACs) data for the expression of a set of relevant genes was available at several time points during the differentiation. From these measurements, summary statistics corresponding to the proportions of cells in each cell type at each time point were used to fit the parameters. Here, a combination of the choice and flip landscape was found to describe the system (§2). The predictive power of the landscape model was demonstrated because simulations from the model accurately predicted the outcome of previously untested experimental conditions.

Adapting this approach to scRNA-seq data remains a challenge because of the high dimensionality and complexity of the data. However, the theoretical underpinnings provide a universal dimension-independent topological structure for attractors, index 1 saddles, and their unstable manifolds (i.e. the decision structure) ([2] electronic supplementary material, appendix A). This suggests a new approach to dimension reduction in which the decision structure and behaviour of a system is given by the topology and motion of a model embedded in a few dimensions that captures the important correlations in the data.

## 6. Biological implications

### 6.1. Waddington dynamics and classifying decision-making

Developmental systems are characterized for being reproducible and robust to noise and insults. These characteristics align with the concept of genericity in mathematical terms, where a property remains when the system is subject to perturbations. Hence, it is natural to assume that the mathematical systems relevant for developmental modelling are generic in these terms. This limits the possibilities to a small list and this list can function as a classification scheme. For dynamical systems with a finite number of rest points (e.g. attractors and saddles) and no other recurrent behaviour (such as chaos), one obtains a clear and detailed picture. Parametrized landscape families have bifurcation sets made up of the components described in §4. The non-bifurcating systems are MS and automatically have a downhill landscape description close to that envisaged by Waddington [1]. This classification scheme describes the bifurcation structure of the relevant GRNs. Taken together therefore, this suggests that it is reasonable to expect that cell decision-making will be represented by one of the small number of decision structures described in this way. These archetypal landscapes provide a conceptual framework for understanding the cell fate decisions and the operation of GRNs.

This theory also highlights some under-appreciated points about cell state transitions. First, it brings to the fore the role of the unstable manifolds of index 1 saddles in determining the escape paths that underlie the transitions. This is relevant even for noise-driven transitions and provides a potential link between experimental data and theoretical models. Second, in addition to local bifurcations, such as the fold, global bifurcations, which include the heteroclinic flip are likely to be important in cell decisions because they change the decision structure. These facilitate certain types of cell fate transitions, such as the proportional allocation of a precursor population into two differentiated cell types. Third, the landscape potential on its own is not sufficient to determine the paths cells take and is therefore inadequate to determine the decision structure or the changes in this structure caused by changing signals. Additional information is needed to accurately represent cell dynamics and we refer to the complete model as *Waddington dynamics* (figure 6).

An important aspect of the approach we describe is the idea that the connections between cell states along which transitions take place correspond to the unstable manifolds of certain saddle points sitting between the system’s attractors. Thus, our discussion includes a precise idea of a *transition state*. This is a distinguishing feature of the approach with strong predictive content. It perhaps provides insight into why evolution has chosen to construct complex cell types by using signals to steer a sequence of transitions between intermediate cell states (i.e. attractors) as a robust strategy rather than some method more akin to using transcription factors as in synthetic cellular reprogramming. One can reasonably argue that in a complex stochastic dynamical system this sequencing is a more natural, robust and flexible strategy.

Although we have focused on systems with a 2D phase space, in higher dimensions cellular decisions are represented by attractors and the saddles with 1D unstable manifolds. This means saddles with higher dimensional unstable manifolds can be ignored with the caveat mentioned in §3.7. Because of this, the decision structures that arise in higher dimensions have the same topology as those in 2D, provided we assume the phase space is bounded and trajectories cross the spherical boundary transversally. This is a non-trivial result about the topology of the unstable manifolds of these saddles which is explained in §3 of [2]). If the topology is known the way the attractors are joined by saddles can be deduced because these unstable manifolds divide the phase space into the basins of attraction of the various attractors.

Moreover, although GRNs are not necessarily gradient systems, MS theory and extension of this work [2,14] indicate that gradient systems defined using a Riemannian metric can be used to provide qualitatively accurate models of them (box 3) and fit to experimental data. This provides a systematic approach to the sort of gene-free modelling first advocated by Corson & Siggia [8,9] and provides parametrized quantitative models that can be used to generate predictions about new experiments [8,10,11].

These considerations indicate that certain types of data will be particularly valuable. In particular, rather than focusing exclusively on end states, data that are sensitive to the sort of dynamical structures guiding the decision-making, such as saddles and bifuractions, are particularly important. This implies the need for temporal data and perturbation experiments that probe key decision points.

### 6.2. Developmental stability, competence, potency and commitment

As well as providing a classification scheme for cell fate decision-making, landscape models also provide insight into several well-established developmental concepts. The Waddington picture (e.g. figure 1) suggests directionality to development is given by the downhill orientation of the landscape. In a landscape family with changing signals, motion would also be downhill. However, as the signals seen by a cell change over time, and these alter the landscape, what is downhill at one time could be uphill at other times. Similarly, stochasticity introduces fluctuations to the landscape and can therefore alter the trajectories that cells take through the landscape. To accommodate this and orientate developmental paths, the observed bifurcations can be used to define a hierarchy for a given temporal signal that orders the bifurcations into the sequence in which they are encountered by a cell. This provides a hierarchical structure to the set of attractors a cell sees. This viewpoint is also relevant to the idea of the potency of a cell. The hierarchical ordering of the bifurcations indicates which fates remain available to a cell located at a specific position within the landscape.

Two cells will undergo the same sequence of developmental decisions if the corresponding paths in parameter space cross the bifurcation set in the same way. This also offers insight into *developmental stability*: a developmental path is stable if all nearby paths intersect the bifurcation set in a qualitatively similar way. A corollary to this is that paths that are close to a cusp are likely to be unstable, as are paths involving a pitchfork bifurcation. The French flag path in figure 9*a* is stable but the symmetry breaking path *γ*_2_ in figure 10*b* is not. Thus, the reproduciblity of development depends on evolving systems that avoid regions close to cusps. On the other hand, in different contexts, the instability near a cusp might be useful. A population of cells in a similar state crossing near a cusp enables the production a population with a mixture of two distinct states. This could be used for patterning into two states or, by combination with stochasticity, for bet hedging.

Landscape models and the relationships between bifurcation sets also provides an illustration for competence—the ability of a cell to respond to an inductive signal by acquiring a specific identity. The fates available for a cell to adopt are indicated by the bifurcation set and the basins of attraction neighbouring its current attractor. A new competence can be acquired if a signal induces a bifurcation that leads to the creation of a new attractor neighbouring the current attractor. For example, starting in the green attractor in *R*_1_ of figure 7 and following the path indicated by *γ*_1_ would result in cells acquiring competence to become the blue cell type, when they crossed the fold curve $B_{A}$. In the *R*_2_ region cells occupying the green attractor would have the competence to adopt the blue cell type but this would not be realized until fold curve $B_{B}$ is crossed.

Furthermore, commitment—the irreversible assignment of a cell to a particular identity—can also be viewed from the perspective of landscape bifurcations. If, following a bifurcation, the landscape returns to its original configuration when the original signalling regime is reinstated, a cell could fall back into its original basin. This will happen if the new attractor disappears when the original signal is reinstated, or if the cell has had insufficient time to cross the saddle. By contrast, given sufficient time after a bifurcation, transitioning cells will have travelled down the unstable manifold towards the new attractor. Reversing the signal in this situation will re-establish the old attractor, but now the cells will be on the other side of the saddle and therefore committed to the new identity. Thus the saddle defines the point of no return and determines when commitment occurs. In this view, therefore, phenomena such as potency, competence and commitment arise out of the dynamical properties of the system and are a natural consequence of the dynamical landscape of cell differentiation. Having this picture in mind should enable the design of experiments that distinguish these topological mechanisms from genetic ones that enforce commitment.

## 7. Outlook

The image of the Waddington landscape has become a powerful and intuitive metaphor for the process of developmental decision-making. Here we have argued that applying techniques from dynamical systems theory offers the opportunity to turn this metaphor into quantitative and predictive models, based on experimental data, that shed light on the underlying biology. Not only are these methods applicable to cells in developing tissues but they can also be used to study the *in vitro* differentiation of stem cells and dysregulated cellular behaviour in diseases such as cancer. To fully realize this potential, further developments are necessary.

To construct a landscape model from experimental data, the first step is the identification of the appropriate family of landscapes. This can be challenging. However, the realization that there are a limited set of simplest qualitatively distinct geometries, each of which has distinct features, provides a framework for designing informative experiments to distinguish between options. After determining the relevant landscape family, building and analysing a parametrized model would be facilitated by methods for embedding high-dimensional data into dynamical models and by statistical methods for fitting models to large high-dimensional data. We have suggested approaches to this (§5), but it is far from clear which methods will be optimal. Another challenge concerns the inclusion of cell–cell signalling into the models. While this is feasible within the framework that has been developed, practical limitations can arise relating to the size and complexity of the resulting simulations. The success in doing this in some studies [8–10] provides a template for future applications but new methods are needed to provide a practical approach for general systems, describing large numbers of cells. Finally, linking the detailed dynamics of the gene regulatory networks to the behaviour described by the dynamical landscape would offer insight into how the logic of developmental decisions are implemented by the underlying molecular mechanisms.

**Figure 13.**
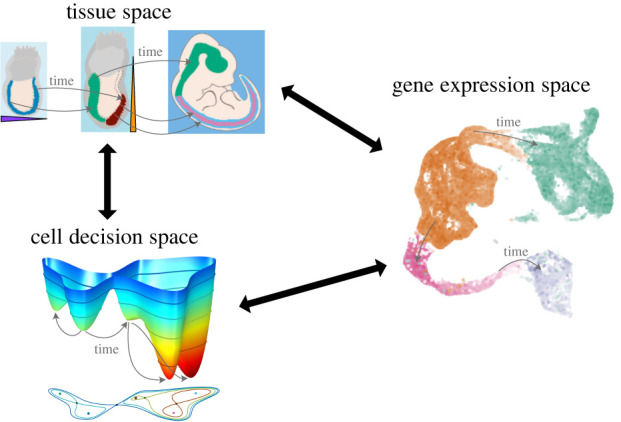
Three developmental spaces. A developing cell can be followed through three distinct spaces. The location of a cell in tissue space gives the coordinates of a cell in a developing embryo and indicates the signalling environment to which it is exposed. The same cell can also be represented in gene expression space by its molecular identity. In addition, the cell occupies a position within cell decision space. This is the location in a Waddington dynamics landscape, which represents its cell fate and describes the decision-making process. The goal is to be able to link cells in these three spaces and to map how a cell moves through them over time.

Ultimately the goal is to connect a cell, located at specific position in a tissue, with its molecular identity, represented in gene expression space, to its fate, represented as its position in a cell decision landscape. These three perspectives provide distinct descriptions of the process of cell decision-making (figure 13). Following the trajectory of a cell as it moves through these three spaces will offer a more comprehensive understanding of how cell fate decisions are made and raises the prospect of being able to control and alter the process with accuracy and precision.

## Data Availability

This article has no additional data.
